# Candidate antigens for serological testing (ELISA) and diagnosis of canine visceral leishmaniasis

**DOI:** 10.1590/S1984-29612025045

**Published:** 2025-10-17

**Authors:** Maria Gabriela Sampaio Lira, Ranielly Araujo Nogueira, Renata Mondêgo-Oliveira, Allana Freitas Barros, Higor da Silva Ferreira, Carla Janaina Rebouças Marques do Rosário, Fábio Henrique Evangelista de Andrade, Flávia Raquel Fernandes do Nascimento, David Soeiro Barbosa, Eduardo Martins de Sousa, Ana Lucia Abreu-Silva, Rafael Cardoso Carvalho

**Affiliations:** 1 Universidade Federal do Maranhão – UFMA, Centro de Ciências Biológicas e da Saúde, Programa de Pós-graduação em Ciências da Saúde, São Luís, MA, Brasil; 2 Instituto Federal de Educação, Ciência e Tecnologia do Maranhão – IFMA, Departamento de Educação, Zé Doca, MA, Brasil; 3 Faculdade Edufor, São Luís, MA, Brasil; 4 Universidade Estadual do Maranhão – UEMA, Centro de Ciências Agrárias, Programa de Pós-graduação em Ciência Animal, São Luís, MA, Brasil; 5 Instituto Oswaldo Cruz – IOC, Laboratório Central de Saúde Pública do Maranhão – LACEN, São Luís, MA, Brasil; 6 Universidade Estadual do Maranhão – UEMA, Centro de Ciências Agrárias, Curso de Medicina Veterinária, Laboratório de Anatomopatologia, São Luís, MA, Brasil; 7 Universidade Federal de Minas Gerais – UFMG, Instituto de Ciências Biológicas, Programa de Pós-graduação em Parasitologia, Belo Horizonte, MG, Brasil; 8 Universidade Ceuma – UNICEUMA, Programa de Pós-graduação em Biociências Aplicada a Saúde, São Luís, MA, Brasil

**Keywords:** Antigens, diagnosis, dogs, recombinant proteins, visceral leishmaniasis, Antígenos, diagnóstico, cães, proteínas recombinantes, leishmaniose visceral

## Abstract

The diagnosis of canine visceral leishmaniasis (CVL) relies on parasitological, molecular methods and serological. However, there are difficulties in using these methods to monitor the disease, and there is a need for more efficient diagnostic alternatives for dogs with suspected visceral leishmaniasis, also using clinical aspects of the animals, which that can be useful for surveillance and control. This study aimed to evaluate the serological diagnostic potential of recombinant antigens in order to improve CVL diagnosis. Disease diagnosis, clinical evaluation, and collection of biological samples for laboratory analysis were performed on dogs from the Tirirical District in São Luís, Maranhão, Brazil. The animals were divided into four groups: clinically suspected dogs for visceral leishmaniasis (VL), subclinical dogs for VL, animals with infections, and healthy animals from an endemic area. Subsequently, the animals were tested for the soluble liver antigen (SLA) antigen and recombinant *Leishmania infantum chagasi* antigens rP2a, rP2b, rP0, rHSP70, rHSP83, rH2A, and rKMP-11 using ELISA. The results demonstrate that the recombinant antigens that presented the best sensitivity, specificity, and high or moderate accuracy according to the Receiver Operating Characteristic (ROC) curves were rP2a, rP2b, and rH2A. These results indicate that rP2a, rP2b, and rH2A proteins are candidates for improving the diagnosis of CVL.

## Resumo

O diagnóstico da leishmaniose visceral canina (LVC) é baseado em métodos parasitológicos, moleculares e sorológicos. No entanto, há dificuldades na utilização desses métodos para o monitoramento da doença, havendo necessidade de alternativas diagnósticas mais eficientes para cães com suspeita de leishmaniose visceral, utilizando também aspectos clínicos dos animais que possam ser úteis para vigilância e controle. Este estudo objetivou avaliar o potencial diagnóstico sorológico de antígenos recombinantes, a fim de melhorar o diagnóstico da LVC. O diagnóstico, avaliação clínica e a coleta de amostras biológicas para a análise laboratorial foram realizados em cães do Distrito de Tirirical, São Luís, Maranhão, Brasil. Os animais foram divididos em quatro grupos: cães clinicamente suspeitos para leishmaniose visceral (LV), subclínicos para LV, animais com outra infecção e animais saudáveis ​​de área endêmica. Posteriormente, os animais foram testados para o antígeno antígeno hepático solúvel (SLA) e antígenos recombinantes de *Leishmania infantum chagasi* rP2a, rP2b, rP0, rHSP70, rHSP83, rH2A e rKMP-11, utilizando-se o teste ELISA. Os resultados demonstraram que os antígenos recombinantes rP2a, rP2b e rH2A apresentaram melhor sensibilidade, especificidade e moderada ou alta precisão, de acordo com as curvas Característica de Operação do Receptor (COR). Esses antígenos indicam que as proteínas rP2a, rP2b e rH2A são candidatas para melhorar o diagnóstico de LVC.

## Introduction

Visceral leishmaniasis (VL) is a systemic parasitic disease caused by the protozoa of the genus *Leishmania*, with *L. infantum chagasi* being the most prevalent in the Americas (Safavi et al., 2021). Transmission usually occurs through the bites of sandflies of the genus *Lutzomyia*, which host the promastigote form of the parasite (van Griensven & Diro, 2019). In vertebrate hosts, the parasite assumes an amastigote form and mainly attacks the tissues of the spleen, bone marrow, liver, and lymph nodes (van Griensven & Diro, 2019).

Domestic dogs are considered the main reservoirs of *L. infantum chagasi* infection in Brazilian states and play a crucial role in establishing the peridomestic cycle of VL in urban, peri-urban, and rural areas (Maia et al., 2018). Therefore, one of the most important approaches to control the occurrence of VL in humans is to diagnose infected dogs (Travi et al., 2018).

Canine visceral leishmaniasis (CVL) can be diagnosed using parasitological, molecular and serological techniques (Silva et al., 2016). However, these methods have different characteristics related to invasiveness, sensitivity, specificity, cost, and feasibility of application in the field, making the diagnosis of CVL complex (Fraga et al., 2016; Magalhães et al., 2017; Solano-Gallego et al., 2017). An ideal diagnostic test should be sensitive, specific, easy-to-use, quantitative, and noninvasive, allowing for repeated sample collection (Solano-Gallego et al., 2011; Quinnell et al., 2013). However, all these characteristics have yet to be achieved in a single test.

Serological techniques are the most widely used for the diagnosis of CVL, as they are easy to use, inexpensive, less invasive, and assist in the detection of circulating anti-*Leishmania* antibodies in the blood (Adams et al., 2012; Silva et al., 2013). However, the sensitivity and specificity of these techniques vary depending on the type, source, purity, and preparation of antigens used (Solano-Gallego et al., 2014; Mendonça et al., 2017).

Among the serological techniques, the Ministry of Health recommends an ELISA kit produced by Bio-Manguinhos/Fiocruz Rio de Janeiro to confirm CVL cases. This kit uses soluble and purified antigens of *L. major* like obtained from *in vitro* culture (SLA) (Coura-Vital et al., 2014). The use of SLA in association with ELISA is common in seroepidemiological studies; however, problems have been reported regarding sensitivity and specificity levels, the possibility of cross-reactivity with sera from dogs infected with etiological agents of other diseases, and failure to identify CVL cases in the subclinical phase (Zanette et al., 2014; Solano-Gallego et al., 2017).

Owing to these potential difficulties, there is a need to improve the existing serological diagnostic methods. Therefore, the use of recombinant *Leishmania* antigens associated with serological techniques has proven to be a valuable and promising alternative because they reduce the occurrence of cross-reactions, provide good sensitivity and specificity, and their production is independent of parasite growth, occurring in a standardized and uniform manner (Celeste et al., 2014). In this context, with the aim of a) avoiding erroneous results in the diagnosis of dogs with VL; b) complementing current diagnostic tools; c) contributing to improving infection control and prevention measures in humans and animals; and d) providing support for constructing a sensitive and specific diagnostic kit for CVL, capable of being applied in the field quickly, the present study used ELISA to determine the diagnostic accuracy of seven recombinant proteins (rP0, rP2a, rP2b, rHSP70, rHSP83, rH2A, and rKMP-11). These proteins were chosen because of their good capacity for recognition by antibodies present in the sera of humans and dogs infected with different species of *Leishmania* (Souza et al., 2013). It is also worth noting that studies on their applicability to sera from dogs infected with *L. infantum chagasi* are scarce or nonexistent in several endemic areas of Brazil, such as the state of Maranhão.

The recombinant proteins rP0, rP2a, rP2b, rHSP70, rHSP83, rH2A, and rKMP-11 are crucial in the study of leishmaniasis, as they offer significant potential for improving the serological diagnosis of the disease. For example, rKMP-11 has demonstrated high reactivity with sera from both humans and dogs infected with *Leishmania* (Carvalho et al., 2003; Iniesta et al., 2002). Additionally, the ribosomal proteins rP2a and rP2b are effective in diagnosis, producing high antibody titers in infected dogs and humans (Soto et al., 1996, 2000). The heat shock proteins rHSP70 and rHSP83 are not only highly conserved but also possess adjuvant properties that enhance the sensitivity of diagnostic assays (Soto et al., 2009). Furthermore, histones such as rH2A have been identified as immunodominant antigens in both humans and dogs (Maalej et al., 2003; Passos et al., 2005), further validating these proteins as key targets for the development of effective diagnostic systems for leishmaniasis.

## Material and Methods

### Study area

The biological samples obtained from the dogs used in the study were collected from the Tirirical District, located in the municipality of São Luís, Maranhão, because of the high seroprevalence of CVL, as demonstrated in previous studies (Abreu-Silva et al., 2008; Barbosa et al., 2010). In this district, four neighborhoods with established transmissions (Cidade Operária, Cidade Olímpica, Jardim América, and São Cristovão) collected samples for animal examination. The samples collected to diagnose the disease were analyzed by considering educational and health strategies and raising awareness among the population through residents' associations or schools in the aforementioned neighborhoods.

### Clinical evaluation and biological samples from dogs

The samples used in this study were obtained from animals whose owners chose to collaborate. A total of 180 samples were obtained from domestic dogs of both sexes of various breeds, between the ages of 6 months and 16 years, collected from the Tirirical District, São Luís, MA. The sample size was calculated according to the guidelines established by the Pan American Center for Zoonoses (Cepanzo, 1979), using the formula for proportion studies, resulting in a total of 180 samples required. Inclusion criteria were as follows: dogs residing in the selected neighborhoods from the Tirirical District and dogs older than six months, due to the disease's incubation period (Brasil, 2014). Of the 180 dogs evaluated, 25.5% (n=46) came from the Cidade Olímpica neighborhood, 25% (n=45) from Cidade Operária, 25% (n=45) from Jardim América, and 24.4% (n=44) from São Cristovão.

A consent form was applied to perform the tests on the animals, which was signed by all dog owners. All experimental protocols involving animals were approved by the Ethics Committee for Animal Use of the State University of Maranhão (approval number 33/2015).

### Clinical, laboratory tests and experimental groups

The dogs were clinically examined by veterinarians and assessed based on the presence of the following clinical signs: lymphadenomegaly, alopecia, skin lesions, lesions on the snout/ears, coat opacity, keratoconjunctivitis, onychogryphosis, cachexia, and weight loss, in addition to parasitological and serological laboratory tests. Animals presenting four or more clinical signs, along with positive parasitological and serological tests, were classified as clinically suspected, while those between zero and three clinical signs were classified as subclinical (Silva et al., 2017). After clinical examinations, blood samples (5 mL) were collected by venipuncture of the cephalic vein or external jugular vein: 4 mL of blood was processed and stored at −20 °C in the Laboratory of Anatomopathology of the State University of Maranhão (São Luís – MA), for serological tests; and 1 mL was used to diagnose *Ehrlichia canis* infection. *E. canis* infection was investigated so that the sera of infected dogs could be used as a specificity control in ELISAs developed using recombinant antigens and SLA.

Serum samples were initially screened with a commercial ELISA/S7® kit (Biogene Indústria e Comércio Ltda) to determine the presence of sera from dogs that were reactive or non-reactive for VL among the samples collected. The ELISA/S7® is based on a recombinant peptide, produced through genetic engineering, which allows the detection of antibodies in the earliest phase of *Leishmania* infection (Andrade & Andrade, 1995). The serological tests were performed according to the manufacturer's protocol. In addition, to confirm seropositive cases from screening, bone marrow aspiration punctures were performed from the iliac crest of the dogs and smeared on a microscope slide. All smears were stained with Panótico Rápido® and examined microscopically for the presence of *Leishmania* amastigotes, at 40× magnification. The dogs were divided into four groups: group 1 (G1), clinically suspected dogs − with four or more clinical signs indicative of CVL positive parasitological and serological laboratory tests for *Leishmania* (n=40); group 2 (G2), subclinical dogs − with up to three clinical signs indicative of CVL and positive parasitological and serological laboratory tests for the parasite (n=47); group 3 (G3), dogs with another infection − carriers of *E. canis* (n=34); and group 4 (G4), healthy dogs from an endemic area − without a diagnosis of infections (n​=59).

### Cross-reactivity

To define possible cross-reactions with ELISAs for the diagnosis of CVL, recombinant antigens and SLA were evaluated against serum samples from dogs infected with *E. canis* (specificity parameter), another common zoonotic species endemic to dogs in the Tirirical District of Maranhão, Brazil.

Complete blood count (CBC) was performed on the dogs' blood samples to check for increased plasma protein levels, thrombocytopenia, anemia, leukopenia, and/or lymphocytosis. Dogs with these CBC results and those not seropositive for VL were positive for *E. canis* when parasitized cells were observed in the blood smear slides.

### Evaluation of recombinant antigens and SLA

The samples examined and screened, containing groups of sera from dogs with clinical (n=40) and subclinical CVL (n=47), sera from dogs with ehrlichiosis (n=34), and healthy dogs (n=59) from the endemic area of ​​the Tirirical District, were used to evaluate the SLA (Souza et al., 2013) and the recombinant antigens rHSP70 (Souza et al., 2013), rHSP83 (Angel et al., 1996), rH2A (Iborra et al., 2004), rKMP-11 (Fuertes et al., 2001), rP0 (Iborra et al., 2003), rP2a and rP2b (Iborra et al., 2007), by ELISA. The following serological assays followed a protocol adapted from Ramírez et al. (2020), using plates previously sensitized with recombinant antigens at a concentration of 1 μg/mL and soluble antigen at a concentration of 2 μg/mL. To begin the tests, the plates were blocked with PBS-Tween 0.5% plus 5% skim milk (200 μL) to prevent non-specific protein binding. After washing, the plates were incubated with the diluted test sera for two hours at room temperature, with 100 μL per well. Each plate included two wells with positive control sera and two wells with negative control sera, both diluted at 1:200 in PBS-Tween 0.5% plus 5% skim milk, in addition to two wells with background control (only blocking solution). Following this, the plates were washed with 200 μL per well of PBS-Tween 0.5%. After the final washing series, the wells were incubated with 100 μL of secondary antibody (anti-canine IgG) conjugated with peroxidase (Sigma Cat No: A6792), diluted at 1:6000 in PBS-Tween 0.5% plus 5% skim milk, and incubated for one hour at room temperature with agitation. For the preparation of the development solution, the contents of one capsule of phosphate-citrate buffer with sodium perborate (PCB) (Sigma Cat No: P-4922) were dissolved in 100 mL of distilled water, and one 20 mg tablet of ortho-phenylenediamine (OPD) was dissolved in 50 mL of PCB, adding 200 μL per well. To stop the reaction, 50 μL of stop solution (3M H2SO4) was added per well, and finally, the plates were read individually at λ = 490 nm using an ELISA reader. Serological assays for each antigen were performed twice, yielding similar results. To determine the optimal *cut-off* points for each antigen, ROC curves were used, based on the values that provided the highest sensitivity and specificity. Serological assays for each plate sensitized with an antigen were performed independently in triplicate.

### Statistical analyses

The results were analyzed using the GraphPad Prism 8.0 software. The data initially underwent the Kolmogorov–Smirnov normality test and were then subjected to Chi-square and Fisher's exact tests to assess the dogs' clinical parameters. Data were considered statistically significant at p < 0.05. The antigens were evaluated based on the *cut-off* values obtained from the ELISA results for each *Leishmania* protein, with sera showing ODs above these *cut-off* values considered reactive for CVL. The *cut-off* value for each antigen in the ELISAs was determined using receiver operating characteristic (ROC) curves, calculated by comparing the reactivity values of serum samples from dogs with VL and serum samples from healthy dogs from the endemic área (Souza et al., 2013). The specific *cut-off* values ​​for each recombinant protein are as follows: SLA (0.21), P2a (0.14), P2b (0.20), H2A (0.33), HSP70 (0.16), HSP83 (0.43) P0 (0.14) and KMP-11 (0.20). To determine the differences between clinical, subclinical, ehrlichiosis-positive, and healthy dogs for each of the antigens used, the OD values ​​obtained in the ELISAs were compared using the Kruskal–Wallis test with Dunn's post-test. ROC curves were constructed to verify the overall accuracy of the ELISA based on the area under the curve (AUC) values, a widely accepted measure for assessing diagnostic accuracy (Martínez Abad et al., 2017). Based on the AUC measurements, it was possible to classify the diagnostic test as worthless (AUC=0.5), with low accuracy (0.5<AUC<0.7), moderate accuracy (0.7<AUC<0.9), high accuracy (0.9<AUC<1.0), and perfect (AUC=1).

## Results

### General profile of dogs and main findings

Of the 180 dogs evaluated, 52.8% (n=95) were male and 47.2% (n=85) were female, with ages ranging from 6 months to 16 years. Regarding breed, 73.3% (n=132) of the dogs had no defined breed, while 26.7% (n=48) had a defined breed, the most frequent being Shih Tzu, Poodle, and Pinscher.

In the screening test via ELISA/S7® kit, confirmed using direct parasitological examination, 48.3% (n=87) of the animals were positive for anti-*Leishmania* antibodies, while 51.7% (n=93) were negative.

Clinical and laboratory clinical evaluation showed that 26.1% (n=47) of the dogs were subclinical, 22.2% (n=40) showed clinical signs of infection, 32.8% (n=59) were considered healthy, and 18.9% (n=34) were diagnosed with ehrlichiosis. The predominant clinical signs observed were lymphadenomegaly (45.5%), onychogryphosis (30%), dull coat (27.2%), skin lesions (22.7%), muzzle/ear lesions (18.3%), alopecia (17.7%), and changes in nutritional status (weight loss and cachexia) (17.7%). Among the signs evaluated, skin lesions (0.022; p < 0.05) and cachexia (0.020; p < 0.05) were most associated with the presence of *Leishmania* infection.

When analyzing seroreactivity by sex, 51.6% (n=49) of male dogs were seroreactive, compared to 44.7% (n=38) of female dogs. Although the seroreactivity was higher among males, the difference was not statistically significant found (0.374; p>0.05) (Table 1).

**Table 1:** Sex, age and breed variables associated with serological results obtained by ELISA/S7^®^ from dogs living in the Tirirical District, São Luís-MA.

**Variable**		**Reagents**		**No Reagents**		**Total**		**p**
		**n**	**%**	**n**	**%**	**n**	**%**	
**Sex**	Males	49	51.6	46	48.4	95	100.0	0.374^*^
	Females	38	44.7	47	55.3	85	100.0	
**Age**	6 months to 2 years	40	44.4	50	55.6	90	100.0	0.508#
	3 to 8 years	35	50.7	34	49.3	69	100.0	
	9 years or older	12	57.1	9	42.9	21	100.0	
**Breed**	With DB^a^	21	43.8	27	56.2	48	100.0	0.502*
	Without DB^a^	66	50.0	66	50.0	132	100.0	

a Defined Breed.

* p-value calculated by Fisher's exact test.

# p-value calculated by Chi-square (χ2) test.

Regarding age groups, the seroreactivity rates were 44.4% (n=40) in young dogs (6 months to 2 years), 50.7% (n=35) in adult dogs (3 to 8 years), and 57.1% (n=12) in elderly dogs (9 years or older). No statistically significant difference was observed among the age groups (0.508; p>0.05) (Table 1).

As for breed, 43.8% (n=21) of dogs with a defined breed were seroreactive, while 50% (n=66) of dogs with no defined breed were seroreactive. This difference was not statistically significant (0.502; p>0.05) (Table 1).

### Recombinant antigens and SLA

**Figure 1:**
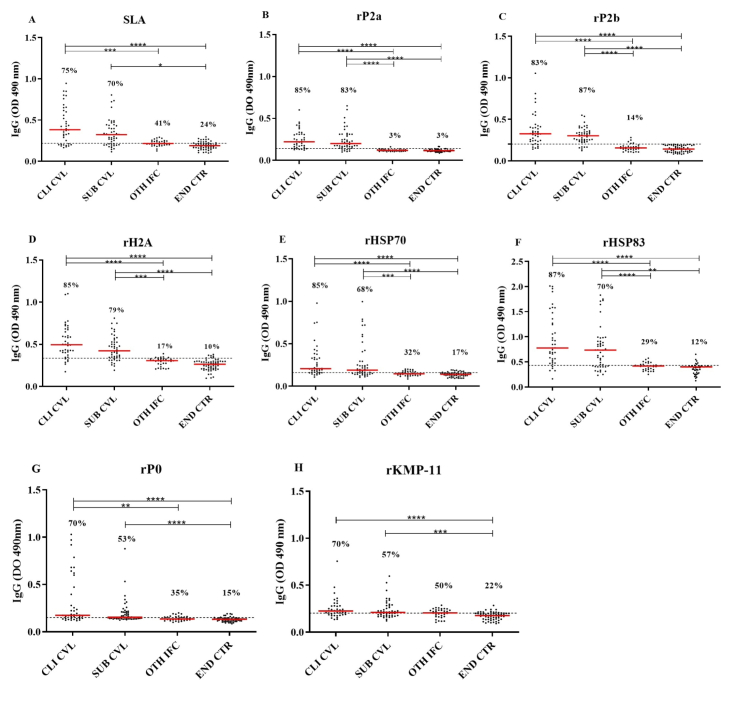
Responses of SLA (A) and rP2a (B), rP2b (C), rH2A (D), rHSP70 (E), rHSP83 (F), rP0 (G), and rKMP-11 (H) antigens to canine sera. Anti-*Leishmania* IgG antibodies in sera from clinical (CLI CVL [n=40]) and subclinical (SUB CVL [n=47]) dogs for canine visceral leishmaniasis (CVL), in dogs with another infection (canine ehrlichiosis) (OTH INF [n=34]) and in healthy dogs from an endemic area (END CTR [n=59]) against SLA, rP2a, rP2b, rH2A, rHSP70, rHSP83, rP0, and rKMP-11 antigens. The points represent the mean of duplicate optical density (OD) values ​​obtained for each canine sample. The dashed lines represent the *cut-offs* established based on the comparison of reactivity values between serum samples from dogs with CVL and serum samples from healthy dogs in the endemic area. The median of the serum groups is represented by the solid red lines. The data obtained for the different serum groups were compared multiple times using the Kruskal–Wallis test with Dunn's post-test. *p<0.05, **p<0.01, ***p< 0.001, ****p < 0.0001.

When evaluating the efficacy of the antigens in recognizing sera from clinical and subclinical dogs for VL, SLA achieved recognition rates of 75% and 70% in sera from clinically suspected and subclinical dogs, respectively (Figure 1A). When recombinant proteins were used, satisfactory performance was observed when testing the rP2a (Figure 1B) (85% clinical and 83% subclinical) and rP2b (Figure 1C) (83% clinical and 87% subclinical) proteins, which showed recognition of the two clinical forms of CVL in a superior manner to SLA (75% clinical and 70% subclinical). Similar to the two ribosomal acidic proteins (rP2a and rP2b), the histone rH2A (Figure 1D) was effective in recognizing clinical (85%) and subclinical (79%) dogs with VL, thus serving as an important candidate for replacing the soluble antigen of *Leishmania*. The heat shock protein antigens rHSP70 (Figure 1E) and rHSP83 (Figure 1F) were efficient in recognizing dogs with clinical form (both had at least 85% sensitivity); however, when considering the sera of subclinical dogs, they obtained similar performance (rHSP83 – 70%) or lower (rHSP70 – 68%) as that of SLA.

The other proteins, rP0 (Figure 1G) (70% of clinical and 53% of subclinical animals) and rKMP-11 (Figure 1H) (70% of clinical and 57% subclinical animals), were not as satisfactory in recognizing clinical and subclinical animals for CVL when compared to the others evaluated and did not achieve superior performance to the soluble antigen.

To demonstrate the effectiveness of the antigens as candidates for improving the serological diagnosis of CVL, the specificity of each antigen was evaluated by verifying its cross-reactivity with sera from dogs infected with ehrlichiosis. When testing serum samples from animals with ehrlichiosis against *Leishmania* antigens, high cross-reactivity with the soluble antigen (41%) was observed (Figure 1A); however, the recombinant protein rKMP-11 was inferior in this regard (Figure 1H) (50% of the sera from dogs with ehrlichiosis). The recombinant heat shock proteins rHSP70 (32%) (Figure 1E) and HSP83 (29%) (Figure 1F), together with the rP0 protein (35%) (Figure 1G), demonstrated moderate cross-reactivity although they showed superior performance to SLA in terms of specificity.

In contrast, proteins rP2a (3%) (Figure 1B) and rP2b (14%) (Figure 1C) were highly specific, with few cross-reactions occurring when tested with sera from dogs with ehrlichiosis and expressed antibody titers with statistically significant differences between the groups of sera used. In addition, the rH2A antigen (17%) (Figure 1D) showed good results in terms of specificity when another type of infection was considered.

Regarding the healthy control dogs from an endemic area, a greater number of false-positive results were observed for the SLA antigen (24%) (Figure 1A) compared to the performance of the recombinant proteins, with emphasis on the ribosomal acidic proteins rP2a (3%) (Figure 1B) and rP2b (0) (Figure 1C), which yielded few or no false-positive results when considering the group of healthy animals from the Tirirical District. Furthermore, it was possible to observe that the differences between the values ​​of peak densities (ODs) of the sera of canines with VL (clinical and subclinical) and healthy dogs from an endemic area were statistically significant for all antigens.

**Figure 2:**
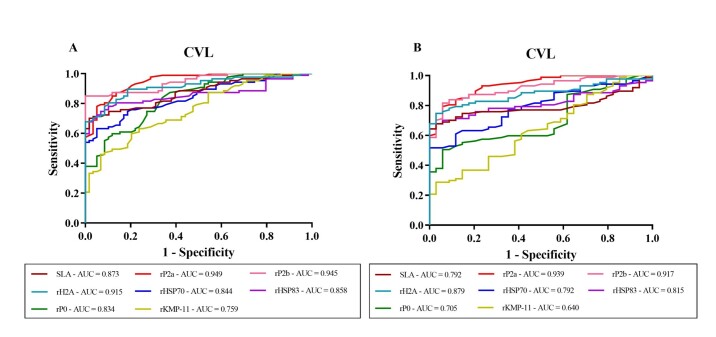
(A) ROC curve of antibody concentrations obtained with the antigens SLA, rP2a, rP2b, and rH2A rHSP70, rHSP83, rP0, and rKMP-11 to detect dogs with VL among healthy animals from an endemic area. (B) ROC curve of antibody concentrations obtained with the antigens SLA, rP2a, rP2b, and rH2A rHSP70, rHSP83, rP0, and rKMP-11 to detect dogs with VL among those infected with another disease. The ROC curve was constructed by comparing the IgG titers of dogs with VL, including clinically suspected and subclinical dogs (n=87). The results of serology tests from healthy dogs from the endemic area of ​​the Tirirical District (n=59) (A) and from dogs with ehrlichiosis (B) were used as a control group. Serology tests were performed against the antigens SLA, rP2a, rP2b, and rH2A. The area under the curve (AUC) is represented by the solid lines, and the dashed line represents the identity of the curve.

Based on the results obtained from ELISAs with SLA and recombinant antigens, ROC curves were constructed to verify the ability of these parasitic antigens to discriminate dogs with VL from healthy dogs in an endemic area. For these ROC curves, all dogs with VL, including clinical and subclinical dogs, were considered. The best performance was observed for the antigens rP2a (AUC: 0.949; p<0.0001), rP2b (AUC: 0.945; p<0.0001), and rH2A (AUC: 0.915; p<0.0001), as they presented high accuracy, with an AUC value higher than that of SLA (AUC: 0.873; p<0,0001) and were able to discriminate between the sera of dogs with VL and healthy dogs from the endemic area of the Tirirical District in a statistically significant manner (Figure 2A). The other recombinant proteins, including rP0 (AUC: 0.834; p<0.0001), rHSP70 (AUC: 0.844; p<0.0001), HSP83 (AUC: 0.858; p<0.0001), and KMP-11 (AUC: 0.759; p<0.0001), exhibited lower AUC values but were still considered satisfactory and statistically significant when compared to SLA.

SLA and the recombinant proteins were again evaluated using ROC curves to determine their performance in discriminating dogs with VL from those infected with *E. canis* (Figure 2B). The antigens that showed the best curve parameter values were rP2a (AUC: 0.939; p<0.0001) and rP2b (AUC: 0.917; p<0.0001), which achieved high accuracy values compared to SLA (AUC: 0.792; p<0.0001). The other recombinant proteins, rH2A (AUC: 0.879; p<0.0001), rHSP70 (AUC: 0.792; p<0.0001), rHSP83 (AUC: 0.815; p<0.0001), and rP0 (AUC: 0.705; p=0.0004), exhibited moderate accuracy, except for KMP-11 (AUC: 0.640; p=0.01635), which showed the worst performance, demonstrating low accuracy, and did not obtain a significant value regarding the specificity parameter.

## Discussion

This study found a prevalence of 48.3% of *Leishmania* infection in the dogs evaluated. Studies in Brazil show significant variation in cases of CVL, with prevalences ranging from 3.8% in Juiz de Fora, Minas Gerais – MG (Castro-Júnior et al., 2014) to 67% in São Luís, Maranhão – MA (Barbosa et al., 2010).

The number of animals without clinical signs sufficiently suggestive of *Leishmania* infection exceeded the number of clinical animals in this study, which is worrying because subclinical dogs are not easily associated with VL but play the same role as clinical dogs in the transmission of the disease, demonstrating the importance of a differential diagnosis in endemic areas. Similarly, in Poxoréu, Mato Grosso do Sul, Azevedo et al. (2008) found that 55.2% of the seropositive dogs with VL were subclinical.

Sex, age, and breed were not associated with seropositivity for CVL. Similarly, two studies conducted in Brazil did not show any sexual predisposition related to infection (Gontijo & Melo, 2004) and Mohebali et al. (2005) reported no significant association between age and disease prevalence in the dogs they evaluated. Belo et al. (2013) conducted a systematic review and meta-analysis to investigate the association between the occurrence of CVL and the age, sex, and breed of dogs. Although no statistically significant association was observed between sex and age, this study indicated a greater probability of *Leishmania* infection in male and older dogs. Belo et al. (2013) revealed that mixed-breed dogs are less likely to be infected than purebred dogs. Other studies have indicated differences in serological responses between mixed-breed and purebred dogs; however, these have been described in Boxer, German Shepherd, Coker, and Doberman dogs (França-Silva et al., 2003; Quilez et al., 2012), which were not among the breeds included in our study.

The signs most associated with serological reactivity to *Leishmania* infection were changes in nutritional status (cachexia) and skin lesions. These and other signs were identified to have a greater association with CVL in the study by Silva et al. (2017), who highlighted the importance of these signs in predicting disease status. All clinical signs observed in the dogs examined have been reported in clinical studies of CVL (Freitas et al., 2012; Manzillo et al., 2013), with an emphasis on skin lesions. Therefore, the clinical signs analyzed should be carefully considered in sick dogs from endemic areas, as they may be indicators of the parasitosis.

As mentioned above, the clinical signs of CVL are quite variable, and the identification of infection, which is mainly based on serological tests, is sometimes difficult. In most cases, infected dogs live in areas endemic for leishmaniasis and are clinically healthy with no signs of disease (Otranto et al., 2009; Paradies et al., 2010). As these infected animals can remain subclinical for long periods, they can be classified as false negatives in clinical trials, which contributes to the maintenance of the parasite transmission cycle (Moreno et al., 2009; Moshfe et al., 2009). Thus, there is a need to improve the current diagnostic tools for CVL based on parameters such as sensitivity, specificity, feasibility, cost, and applicability in the field. In this context, a growing number of recombinant proteins have been described as alternatives to the SLA, which is used in the Biomanguinhos enzyme-linked immunosorbent assay recommended by the Ministry of Health for the serodiagnosis of VL in dogs (Souza et al., 2012; Rafati et al., 2006). The use of soluble antigens in the diagnosis of CVL can cause cross-reactions with sera infected by etiological agents of other diseases, such as ehrlichiosis, babesiosis, and neosporosis (Celeste et al., 2004; Zanette et al., 2014). Several recombinant proteins have been tested to overcome such nonspecific reactions (Celeste et al., 2004; Arruda et al., 2013).

In this study, ELISA was performed to validate the performance of recombinant antigens of *L. infantum chagasi* in recognizing sera from dogs with and without VL symptoms. In general, all recombinant proteins evaluated in this study were recognized by sera from dogs with and without VL symptoms, although with different antibody titers. Among these, rP2a, rP2b, and rH2A showed the best performance in terms of sensitivity and were superior to SLA in recognizing *Leishmania* infection. The antigenicity of ribosomal acidic proteins, mainly rP2a and rP2b, has already been analyzed in certain studies against sera from dogs and humans infected with VL, demonstrating recognition between 80% and 100% by the sera (Soto et al., 2009; Carrillo et al., 2008; Iniesta et al., 2008), This was the case in this study, with two subunits of ribosomal acidic proteins (rP2a and rP2b) that can recognize a large number of clinical and subclinical dogs. Nucleosomal histones have been widely used in ELISA of sera from humans and dogs with VL. Maalej et al. (2003) demonstrated 100% histone rH2A for the detection of sera with VL. Souza et al. (2013) found that histones were recognized by a moderate percentage of *Leishmania*-infected sera, and among the four recombinant histones tested (rH2A, rH2B, rH3, and rH4), rH2A showed the best recognition. In Spain, Soto et al. (1995) indicated that 78% of sera from dogs with VL had significant titers of anti-H2A antibodies. Soto et al. (1998) analyzed histone rH2A in sera from dogs with leishmaniasis and found recognition of 72% of the infected sera, surpassing the SLA, similar to the data observed in this study.

Heat shock proteins (rHSP83 and rHSP70) showed good sensitivity in recognizing sera from clinical and subclinical dogs with VL. Several authors have reported that heat shock proteins are notable during infection by *Leishmania* spp., and their antigenicity for sera with the disease is not unusual (Carrillo et al., 2008; Celeste et al., 2004). In the study by Andrade & Andrade (1995), the recombinant antigen rHSP70 demonstrated good sensitivity parameters when analyzed against sera with VL, obtaining good sensitivity performance.

Celeste et al. (2004, 2014) investigated the rHSP83 protein of *L. infantum* using serum samples from human patients with cutaneous leishmaniasis and VL and found a sensitivity of 100%. Approximately 90% of sera with CVL are recognized by the antigen, confirming that it is a recombinant protein useful in the serodiagnosis of canine leishmaniasis (Skeiky et al., 1995). Menezes-Souza et al. (2014) tested the heat shock antigen rHSP83 with sera from dogs with VL and obtained satisfactory performance in the diagnosis of *Leishmania* infection, as demonstrated in this study for both clinically suspected and subclinical dogs.

The ribosomal acidic protein rP0 has been recognized in the sera of humans and dogs infected with *L. infantum* (Soto et al., 1995, 2009). Soto et al. (1995) found that 78% of sera from dogs infected with *L. infantum* contained antibodies reactive to the rP0 protein. In this study, satisfactory results were not found with this antigen regarding the recognition of sera from clinical and subclinical dogs for VL when compared to other recombinant proteins, and it showed a lower performance than SLA and other members of the ribosomal acidic proteins (rP2a and rP2b). However, the rP0 protein has a C-terminal region that is different from those of other ribosomal proteins (Soto et al., 2009), which may have resulted in the different responses observed for ribosomal acidic proteins. The rKMP-11 antigen obtained recognition of clinical and subclinical dogs for VL, similar to the rP0 protein, being lower than that of SLA and other recombinant proteins. A result similar to that demonstrated in this study was verified by Iniesta et al. (2002) and Carvalho et al. (2003), who demonstrated moderate performance in terms of recognition of this antigen by sera from dogs and humans with VL.

SLA showed high cross-reactivity with sera from animals infected with *E. canis*. According to Zanette et al. (2014), techniques that use total antigens are limited in terms of specificity, as they present cross-reactions not only with other species of the Trypanosomatidae family but also with phylogenetically distant organisms. Zanette et al. (2014) verified the occurrence of possible cross-reactions between *E. canis*, *B. canis*, and *L. chagasi* using ELISA. Similarly, Marcondes et al. (2011) evaluated cross-reactivity between *Leishmania* and etiological agents causing Chagas disease, ehrlichiosis, babesiosis, toxoplasmosis, and neosporosis, and found false-positive results. Specifically, 3/6 dogs were seropositive for *Toxoplasma gondii*, 1/2 dogs were seropositive for both *Toxoplasma gondii* and *Neospora caninum*, 3/4 dogs were seropositive for *Babesia canis* and *E. canis*, and 4/7 dogs were seropositive for *Trypanosoma cruzi*. The SLA exhibited high cross-reactivity with sera from animals infected with *E. canis*.

The high cross-reactivity between some antigens and sera from dogs with ehrlichiosis compromises the diagnostic accuracy of the test, as it increases the occurrence of false-positive results and acts as an obstacle in locations where at least two parasites are endemic (Souza et al., 2013). This fact was evidenced in our study by the AUC values ​​in the ROC curves, which is a cause for concern since ehrlichiosis, in addition to being a disease in which dogs normally present clinical signs that resemble those observed in CVL; the two diseases coexist in several regions of the country.

rKMP-11 has been observed to recognize high antibody titers in sera infected with diseases other than leishmaniasis, especially when analyzed in sera from humans with Chagas disease (Souza et al., 2013). As in the literature, the data demonstrated here also show high cross-reactivity between sera from dogs with VL and ehrlichiosis, superior to SLA and other recombinant proteins (rP0, rP2b, rHSP70, rHSP83, and rH2A), which limits the performance of rKMP-11 in the diagnosis of dogs clinically suspected or subclinical for leishmaniasis.

The recombinant proteins rP2a and rP2b did not cross-react with sera from dogs with ehrlichiosis, and false-positive results with sera from healthy dogs were low (rP2a = 1/34; rP2b = 0/34), resulting in high accuracy values ​​when evaluated using ROC curves. However, in addition to being recognized by sera from dogs and humans with leishmaniasis, ribosomal acidic proteins have also shown moderate recognition of sera infected by etiological agents that cause other infectious diseases (Soto et al., 2009; Levin et al., 1993), differing from the findings of this study, in which the level of cross-reactivity was insignificant.

Furthermore, in the research carried out by Soto et al. (1996), in which they tested the combination of rP2a and rP2b proteins (chimeric antigen) against sera with leprosy, tuberculosis, and Chaga disease, no cross-reactivity was observed, indicating that the performance of recombinant antigens can be increased when used in combination with other *Leishmania* antigens and, thus, can meet the requirements for a specific diagnostic tool capable of differentiating diseases that may share some clinical manifestations.

The heat shock protein, rHSP70, performed similarly to SLA in terms of specificity, showing cross-reactivity with sera from dogs with ehrlichiosis. Zurita et al. (2003) evaluated the recombinant antigen rHSP70 and found that in addition to being recognized by sera with VL, it was also recognized by sera infected with *Trypanosoma cruzi*. The cross-reactivity observed for the antigen rHSP70 demonstrates that its antigenic determinants are not restricted to the recognition of *Leishmania* infection, as high to moderate levels of cross-reactivity have been demonstrated.

The rHSP83 antigen, although not yet analyzed, has been investigated in serum samples with cutaneous leishmaniasis and visceral leishmaniasis, with a specificity of 100% observed when tested against serum samples from individuals with Chagas disease (Celeste et al., 2004), unlike the results observed for CVL, in which moderate cross-reactivity was found with sera from dogs infected with *E. canis*.

The rP0 protein cross-reacts with sera from patients with chronic autoimmune diseases and Chagas disease (Soto et al., 1995). Skeiky et al. (1995) analyzed three subunits of ribosomal acidic proteins, rP0, rP2a, and rP2b, and found that sera infected with *T. cruzi* reacted exclusively with rP0 but not with rP2a and rP2b proteins. Our results also demonstrated greater cross-reactivity between the rP0 protein and sera from dogs with ehrlichiosis, which was different from that of the other ribosomal acidic proteins (rP2a and rP2b) tested in this study, perhaps because of the divergence of peptides that make up the C-terminal sequence, which may have disadvantaged the rP0 protein.

Concerning histone rH2A, Soto et al. (1992) found moderate cross-reactivity with other infections and diseases. Souza et al. (2013) evaluated the rH2A antigen and found moderate cross-reactivity with sera from patients with diseases other than leishmaniasis. Maalej et al. (2003) reported a specificity of 91% for this antigen. Furthermore, Passos et al. (2005) demonstrated that the ELISA test sensitized with rH2A antigen presented 100% specificity when analyzing cross-reactivity with *T. cruzi*-infected sera. This performance is superior to that obtained in this study, demonstrating the good diagnostic capacity of the antigen for leishmaniasis detection.

## Conclusions

Considering the need for an efficient diagnostic test for CVL, the promising importance of recombinant proteins as diagnostic tools, and the limited number of studies on these recombinant proteins for the detection of leishmaniasis in different endemic areas in Brazil, the findings of this research point to a promising perspective. Of the recombinant antigens evaluated, rP2a, rP2b, and rH2A played excellent roles in the serodiagnosis of CVL, both in terms of sensitivity and specificity, and could detect the disease even in dogs with an subclinical profile, thus becoming important candidates for improving the diagnosis of the disease. In addition, this research opens up prospects for the construction of a sensitive and specific diagnostic kit from a combination of these proteins, which can be used quickly in the field, contributing to the effective and differential diagnosis of clinical and subclinical forms of CVL, especially in endemic areas.

## Data Availability

Data will be made available on request.
